# Association of cataract extraction and the risk of dementia—A systematic review and meta-analysis

**DOI:** 10.3389/fnagi.2023.1168449

**Published:** 2023-05-25

**Authors:** Qiao Zhang, Yuan Ju, Wei Zheng, Lulu Xie, Xi Wang, Huanhuan Ren, Zhipeng Chen, Xingtong Liu, Xiaolin Bai, Ruile Fan

**Affiliations:** ^1^College of Traditional Chinese Medicine, Changchun University of Chinese Medicine, Changchun, China; ^2^Department of Ophthalmology, Affiliated Hospital of Changchun University of Chinese Medicine, Changchun, China

**Keywords:** cataract, dementia, Alzheimer's disease, meta-analysis, cohort study

## Abstract

**Objectives:**

This research aims to investigate if cataract extraction lowers the risk of all-cause dementia.

**Methods:**

Original literature on cataract surgery associated with all-cause dementia as of November 27, 2022, was searched in several commonly used databases. Manual review was used to include eligible studies. Stata software (version 16) was used to perform statistical analysis on pertinent data. Publication bias can be precisely evaluated using funnel plots and Egger's test.

**Results:**

In the meta-analysis of 4 cohort studies with 245,299 participants. Pooled analysis indicated that cataract surgery was linked to a lower incidence of all-cause dementia (OR = 0.77, 95%CI: 0.66–0.89; *I*^2^= 54.7%; *P* < 0.001). Cataract surgery was linked to a lower risk of AD (OR = 0.60, 95%CI: 0.35–1.02; *I*^2^= 60.2%; *P* < 0.001).

**Conclusions:**

Cataract surgery is linked to a lower incidence of all-cause dementia and Alzheimer's disease. A cataract is a reversible visual impairment. Cataract surgery may be a protective factor against the onset of all-cause dementia and can reduce the economic and family burden caused by all-cause dementia worldwide. Given the restricted pool of included studies, our findings necessitate meticulous interpretation.

**Systematic review registration:**

http://www.crd.york.ac.uk/prospero retrieve registration details by searching CRD4202379371.

## Introduction

Dementia is an aging-related illness that causes behavioral and cognitive deterioration in older adults. It has a tremendous impact on individuals, families, and society's economy, with an estimated yearly global cost of around 1 trillion dollars, and an estimated around 50 million individuals worldwide having dementia. As the world's population ages, by 2050, that figure is expected to rise to 152 million (Livingston et al., 2020). Although there are some treatable dementias, such as norm isobaric hydrocephalus (Liang and Chebrolu, 2022) and chronic subdural hematoma (Igami et al., 2022). There are still many dementia patients who currently lack effective treatment methods. Therefore, it has become a top priority for public health to find additional risk factors that can be avoided to stop or delay dementia (Ma et al., 2022). Numerous prospective studies have discovered that sensory deficiencies, such as vision or hearing loss, are each separately linked to an increased risk of dementia (Lin et al., 2013; Fischer et al., 2016; Schubert et al., 2017; Brenowitz et al., 2019). Early studies (Reischies and Geiselmann, 1997; Busse et al., 2002) and population-based epidemiological studies (Anstey et al., 2001; Gussekloo et al., 2005; Valentijn et al., 2005; Tay et al., 2006) have demonstrated that visual impairments and cognitive function are significantly correlated.

Accumulating evidence suggests that sensory problems like hearing loss and vision impairment raise the risk of dementia, and the high prevalence of sensory diseases is particularly notable (Chen et al., 2017; Nagarajan et al., 2022). A cataract is a widespread eye disease, that often affects the vision of the elderly. Over 35 million individuals worldwide are affected by cataracts, which result in blindness in about 20 million of those people (Lee and Afshari, 2017). Cataract extraction and IOL (IntraOcular Lenses) implantation are the most effective treatment for cataracts. With the continuous advancement of medical technology, modern cataract surgery is characterized by high efficacy and low risk that offers patients much value and a 4,500% return on health investment to society, which is higher than other eye disorders (Brown et al., 2014). It is well known that many kinds of eye diseases can cause permanent damage to vision. A cataract, as a reversible visual impairment, could be a potential prevention target with significant global public health and economic impact if cataract surgery can be proven as a protective factor for the risk of developing dementia.

Previous studies have reported that cognitive function improved significantly after cataract surgery, especially in the elderly (Tamura et al., 2004; Gray et al., 2006; Ishii et al., 2008). However, there are also inconsistent and ambiguous research results on whether cataract surgery can reduce the risk of dementia (Grodstein et al., 2003; Tamura et al., 2004; Gray et al., 2006). Therefore, this study aims to conduct a systematic review and meta-analysis of the included literature to ascertain whether cataract extraction plays a significant role in decreasing the incidence or delaying the progression of all-cause dementia.

## Methods

The Preferred Reporting Items for Systematic Reviews and Meta-Analyses (PRISMA) guidelines were followed for this meta-analysis (Page et al., 2021). The protocol was sent to the International Prospective Register of Systematic Reviews (PROSPERO) with the Approval Number CRD42022379371.

### Search strategy

Without language restrictions, we thoroughly searched the Cochrane Library, Embase, and PubMed databases from their establishment until November 27, 2022. Following are the keywords and Medical Subject Headings (MeSH) terms that were utilized in the search:(((“Dementia”[Mesh]) OR (“Alzheimer Disease”[Mesh])) OR (((Dementia^*^[Title/Abstract]) OR (Amentia^*^[Title/Abstract])) OR (Alzheimer^*^[Title/Abstract]))) AND ((“Cataract Extraction”[Mesh]) OR (((((Cataract Extraction^*^[Title/Abstract])) OR (Phakectom^*^[Title/Abstract])) OR (Enzymatic Zonulolys^*^[Title/Abstract])) OR (Cataract Surgery[Title/Abstract]))). The references of the included studies and the already published systematic reviews were searched to locate more pertinent literature. The complete search strategy is included in Supplementary Table 1.

### Eligibility criteria

Include eligible research according to the following requirements: (1) The risk of all-cause dementia is the primary observation indicator, and AD is the secondary observation indicator, expressed as an adjusted odds ratio (OR); (2) cohort, cross-sectional, or case-control study design; (3) The observation group is the patients who have undergone cataract surgery, while the control group is the patients without surgery. All forms of dementia, including those caused by vascular dementia, senile dementia, and Alzheimer's disease, were included in this study.

### Exclusion criteria

(1) Conference abstracts or study protocols, (2) publications containing the same content twice, and (3) studies with insufficient data or uninteresting results.

### Data extraction

Two researchers independently extracted data according to the inclusion and exclusion criteria, and the results are as follows: The first author, publication year, nation, sample size, follow-up years, age, diagnosis of cataract/dementia, outcomes types, confounders adjusted and NOS scores are among the variables that are extracted. When there was a difference of opinion, conversations with a third researcher (JY) were held until an agreement was established.

### Risk-of-bias assessment

Using the Newcastle-Ottawa Quality Assessment Scale (NOS), we assessed the included studies' quality from three perspectives: selection, comparison, and outcome (cohort study) or exposure (case-control study). Cohort and case-control studies received ratings in the range of 0–9. Higher scores suggest higher study quality, with NOS scores of 7 or higher indicating high study quality, 4–6 indicating medium study quality, and 0–3 indicating low study quality.

### Statistical analysis

The data analysis was carried out using Stata software (16). To determine the risk of all-cause dementia following cataract surgery, we gathered information on the adjusted OR and 95% CI from each trial. We used the chi-square test and *I*^2^-value to determine the degree of heterogeneity, and we defined heterogeneity as *p* < 0.1 or *I*^2^ > 50%. A fixed effect model was used for heterogeneity of < 50%. Otherwise, a random effect model was used. Publication bias can be precisely evaluated using funnel plots and Egger's test.

## Results

### Search results

**Figure 1:**
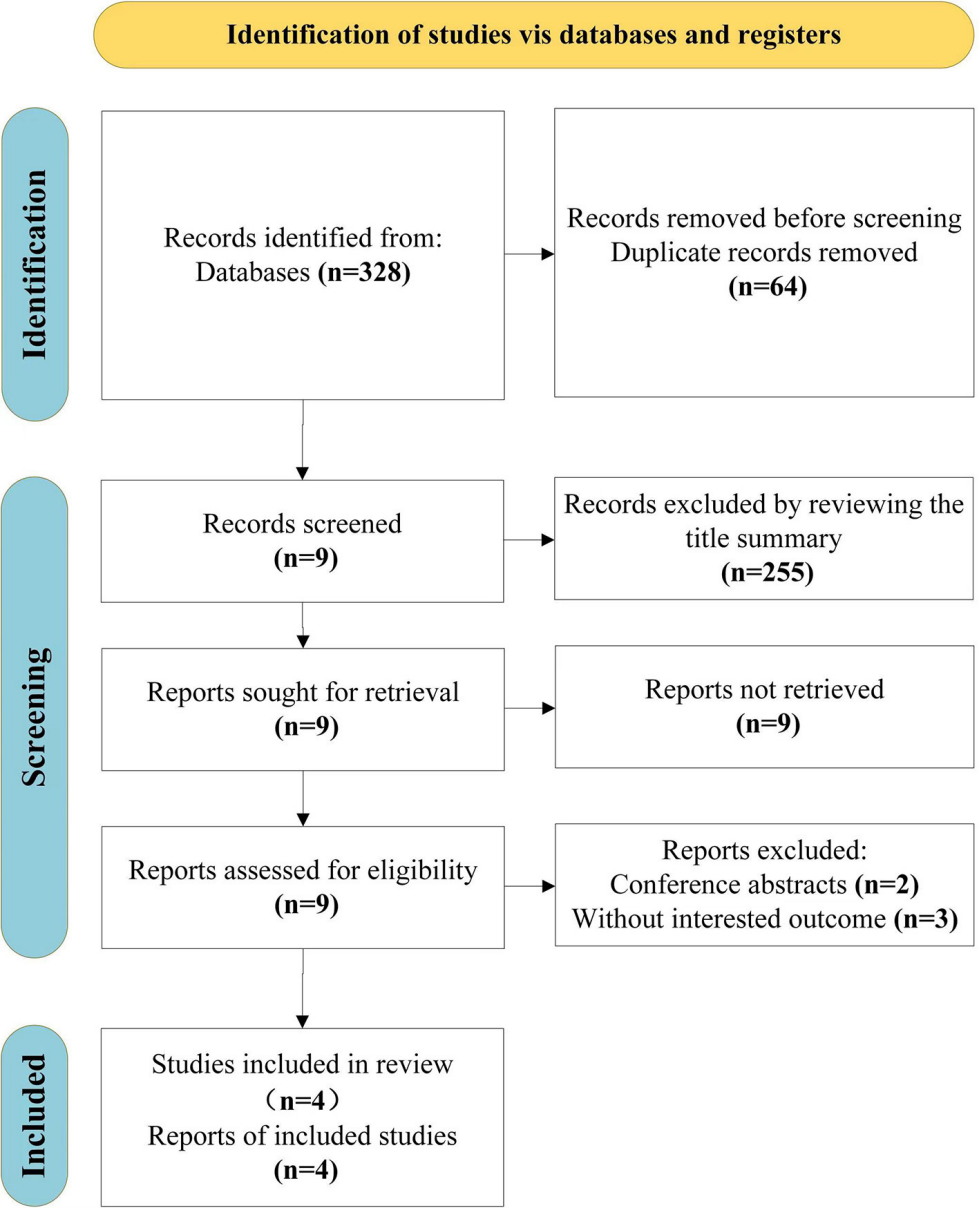
Literature screening flowchart.

From the original search, we found 328 items in total, 64 duplicates we had to remove. After screening the titles and abstracts and the entire texts of 4 publications, including conference abstracts (*n* = 2) and articles with uninteresting results (*n* = 3), 255 unrelated articles were also eliminated. Finally, this review includes four studies (Yu et al., 2015; Miyata et al., 2018; Lee et al., 2022; Ma et al., 2022). The search selection process is shown in Figure 1.

### Study characteristics

A total of 245,299 people were involved in the four trials we included (Yu et al., 2015; Miyata et al., 2018; Lee et al., 2022; Ma et al., 2022). In these investigations, one study (Yu et al., 2015) was a retrospective cohort study, while three studies (Miyata et al., 2018; Lee et al., 2022; Ma et al., 2022) were prospective cohort studies. Between 2015 and 2022, these papers were published. Each study had a sample size of 3,038–226,226 people with a follow-up time of between 8.4 and 10 years. In addition to one investigations (Lee et al., 2022) carried out in the US, others were done in the UK (Ma et al., 2022), Japan (Miyata et al., 2018), and China (Yu et al., 2015). The included studies' participants' mean ages ranged from 40 to 70 years. Three studies (Yu et al., 2015; Lee et al., 2022; Ma et al., 2022) employed the International Classification of Diseases-9 (ICD-9) or International Classification of Diseases-10 (ICD-10) diagnostic codes as the diagnostic criteria for dementia and cataract; the outcome measure was All-cause dementia in four studies (Lee et al., 2022; Ma et al., 2022; Miyata et al., 2018; Yu et al., 2015). The main characteristics of the included studies are shown in Table 1.

**Table 1:** Basic characteristics of the included studies.

**References**	**Country**	**Follow up years**	**Age**	**Sample size (cataract surgery/ no surgery)**	**Diagnosis of cataract**	**Diagnosis of dementia**	**Outcomes types**	**Confounders adjusted**	**NOS scores**
Lee et al. (2022)	USA	10	≥65y	1,382/1,656	ICD-9/10 and CPT codes	DSSMD (fourth edition)	ACD, AD	Education, self-reported White race, smoking, APOE ε4, sex, age groups at cataract diagnosis, diabetes, systolic blood pressure, hypertension, heart disease, cardiovascular disease, BMI, self-rated health, CCI, number of activities of daily living limitations, At least 15 min of activity 3 times/wk, performance-based physical function scores, Center for Epidemiologic Studies Depression Scale scores, retirement status, and self-reported difficulty with distance or near vision.	9
Ma et al. (2022)	UK	8.4	40–69 y	4,281/8,970	ICD-9/ICD-10	ICD-9/ICD-10	ACD, AD, VaD	Age, sex, education, APOE ε4, history of other types of eye surgery, unilateral or bilateral cataracts, cataract duration, ethnicity, body mass index, smoking status, alcohol use, and CVD	9
Miyata et al. (2018)	Japan	/	≥68 y	668/2,096	A landolt ring chart, the slit-lamp examination	The mini-mental state examination (MMSE)	ACD MCI	Age, sex, body mass index, education, BCVA, hypertension, diabetes, depression, and stroke.	8
Yu et al. (2015)	China	10	≥70 y	113,123/ 113,123	ICD-9	ICD-9	ACD	Age, sex, CCI score, the interval between the first coding of cataract diagnosis and index date, hypertension, and diabetes mellitus	7

### Quality assessment

The average score for all included studies was 8.25 under the NOS criteria. Each trial received a score of 7 or above, indicating that all of the studies included in this meta-analysis were high caliber. The scores of the included studies are shown in Table 1.

### Cataract surgery and risk of all-cause dementia

**Figure 2:**
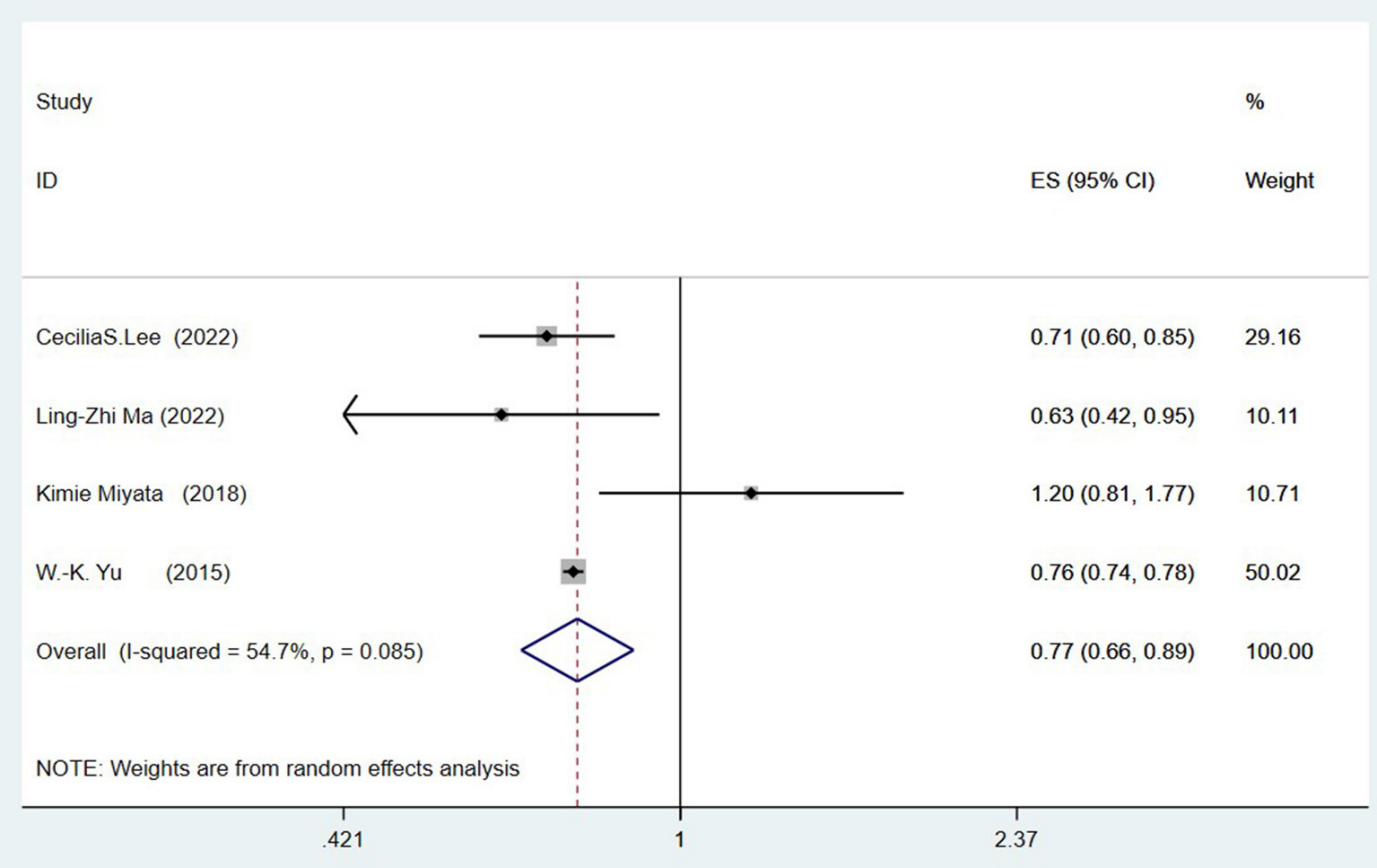
Meta-analysis of cataract surgery and the risk of all-cause dementia.

Four studies (Yu et al., 2015; Miyata et al., 2018; Lee et al., 2022; Ma et al., 2022) explored the link between prior cataract surgery and the risk of all-cause dementia. Combined data revealed that a history of cataract surgery is associated with a reduced risk of ACD (OR = 0.77, 95%CI: 0.66–0.89; *I*^2^= 54.7%; *P* < 0.001; Figure 2).

### Cataract surgery and risk of Alzheimer's disease

**Figure 3:**
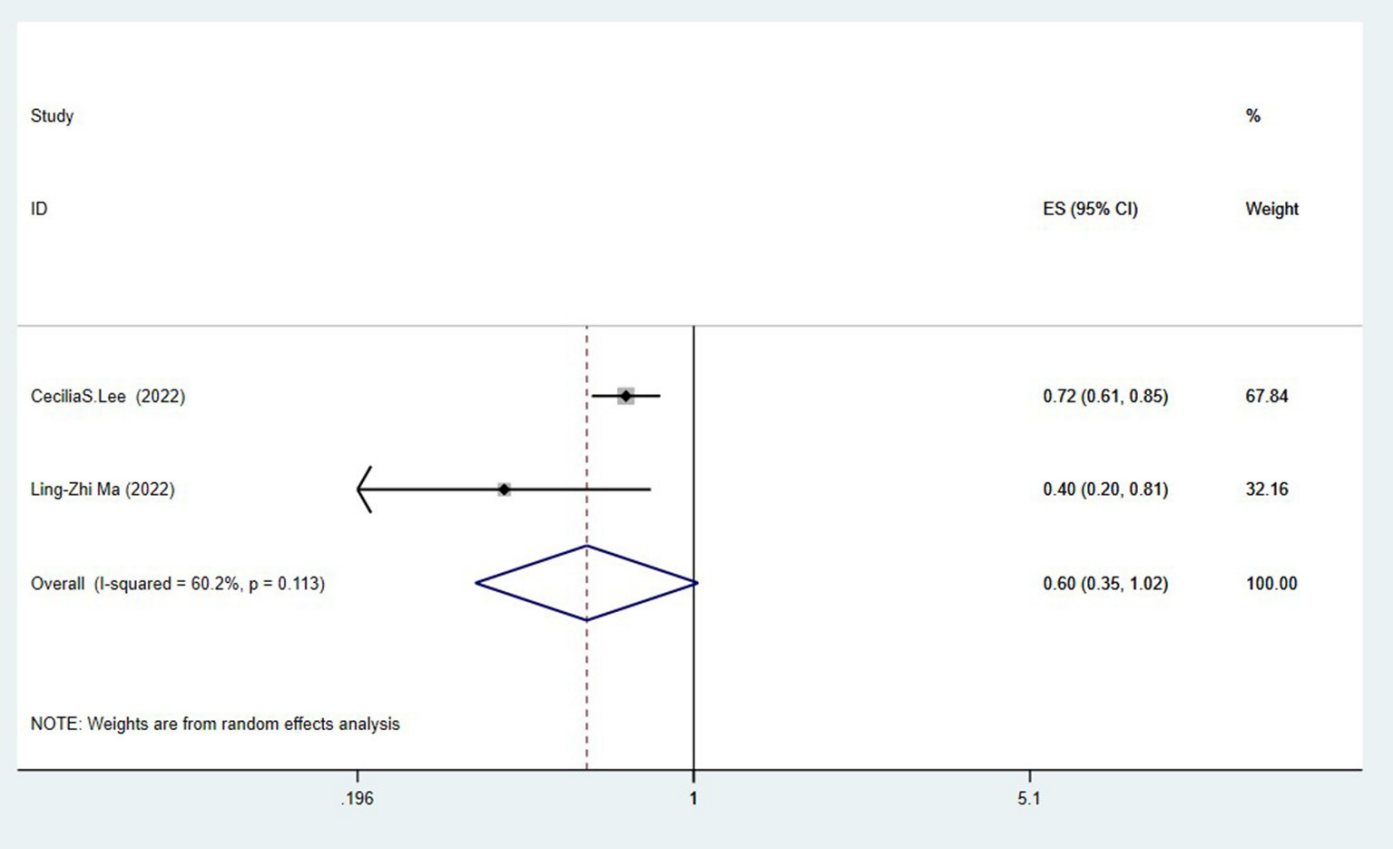
Meta-analysis of cataract surgery and the risk of Alzheimer's disease.

Two studies (Lee et al., 2022; Ma et al., 2022) assessed the association between cataract surgery and the risk of Alzheimer's disease. The pooling analysis found that cataract surgery was related to a reduced risk of Alzheimer's disease (OR = 0.60, 95%CI: 0.35–1.02; *I*^2^= 60.2%; *P* < 0.001; Figure 3).

### Publication bias

**Figure 4:**
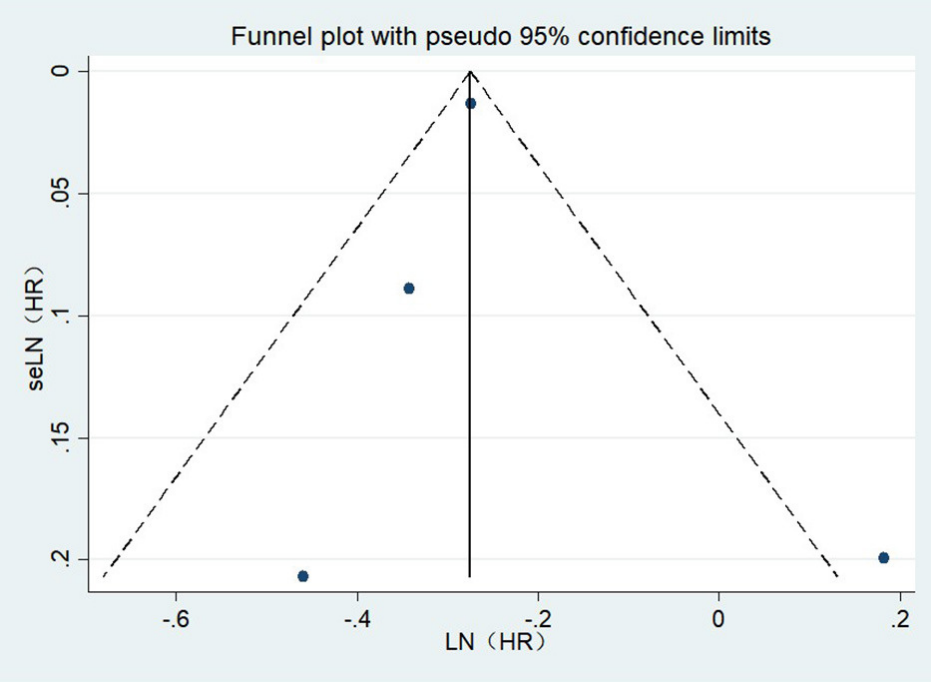
Publication bias of cataract surgery and the risk of all-cause dementia.

A visual examination of the funnel plot revealed no significant publication bias in the outcome of cataract surgery and risk of all-cause dementia (Figure 4). Egger's regression test (*P* = 0.835) also indicated that our meta-analysis had no publication bias.

## Discussion

Our meta-analysis was based on observational, and 4 cohort studies (Yu et al., 2015; Miyata et al., 2018; Lee et al., 2022; Ma et al., 2022) with 245,299 individuals were included to investigate the association between cataract surgery and all-cause dementia. We discovered that cataract surgery significantly reduced the risk of all-cause dementia and Alzheimer's Disease compared to the controls without surgery. Since the etiology of different types of dementia varies, cataract surgery is not meant to reduce all types of dementia. The possible effects of cataracts and their surgical treatment may vary depending on the type of dementia.

Recent meta-analyses (Vu et al., 2021) have suggested that visual impairment may increase the risk of dementia and cognitive impairment and may help to reduce the incidence of dementia through early prevention and timely treatment of visual impairment. This is the same as our current study: Cataract is a kind of treatable eye disease with visual impairment. Cataract with phacoemulsification, and IOL implantation is a highly effective and technically mature procedure; most patients, especially those without other fundus diseases, have improved visual acuity and improved contrast sensitivity after surgery (Powe et al., 1994; Behndig et al., 2011). One study (Miyata et al., 2018) showed no apparent association between cataract surgery and risk of all-cause dementia (OR = 1.20, 95% CI: 0.81–1.77, *P* = 0.36) even after adjusting for many confounders, but may help lower the chance of acquiring Mild Cognitive Impairment (MCI) (OR = 0.79, 95% CI: 0.64–0.97, *P* = 0.025). The following may be the related reasons: MCI, defined as the transition from normal cognition to an early stage of dementia, is a treatable condition (Koepsell and Monsell, 2012). Moreover, the exclusive reliance on MMSE as a diagnostic tool for mild cognitive impairment and dementia in this investigation is insufficient in accurately identifying the disorders. This may also be one of the reasons why cataract surgery does not have a significant inhibitory effect on dementia. Furthermore, no follow-up of the study results was performed, which may have impacted on the final results. One study (Ma et al., 2022) showed no apparent association between cataract surgery and the risk of Vascular disease (VaD) (OR = 0.954, 95% CI: 0.482–1.888, *P* = 0.892), compared to AD. The author in this study noted that cataract surgery alone does not significantly reduce the risk of dementia if the cataract is complicated by systemic disease. Studies (Jaul and Meiron, 2017) have pointed out that the key risk factors for VaD are (1) vascular aging, (2) cardiovascular risk factors, and (3) cerebrovascular risk factors. The process of vascular aging increases the risk of systemic pathophysiological processes (arteriosclerosis) as well as the danger of cerebrovascular injury. This may be the reason that cataract surgery was not significantly associated with VaD. AD and visual impairment share common pathogenesis, and AD patients are more likely to benefit from cataract surgery. The pathogenesis of AD and extracellular amyloid protein β (A β) Sedimentation, oxidative stress, and inflammation are related (Giasson et al., 2002). According to research, the pathophysiology of dementia and visual impairments, particularly Alzheimer's disease and age-related macular degeneration (a frequent age-related visual impairment), is comparable. The development of AMD is influenced by A β, oxidative stress and inflammatory mechanisms (Dentchev et al., 2003; Mandas et al., 2014). Two studies (Yu et al., 2015; Lee et al., 2022) on cataract extraction to reduce the risk of all-cause dementia found through follow-up that this low risk was more obvious in the first 5 years after cataract surgery, suggesting that the long-term benefits of cataract surgery are lasting and effective.

So far, the exact mechanism of the relationship between cataract surgery and dementia has not been fully elucidated, but several hypotheses exist. Older adults with visual impairment are relatively less inclined to participate in social and physical activities and are more likely to suffer from depression, which makes them more susceptible to cognitive impairment (Fischer et al., 2009; Jin et al., 2019; Harithasan et al., 2020). Several studies have showed that social isolation and a lack of cognitive stimulation brought on by sensory impairment may raise the development of dementia (Whitson et al., 2018; Livingston et al., 2020). Reduced central sensory pathway activation from the loss of visual information may raise the danger of cognitive strain and harm intrinsic brain structures (Rutherford et al., 2018; Whitson et al., 2018). These ultimately accelerates the process of cognitive deterioration. According to some research, vision impairment is one of the earliest signs that dementia is developing (Javaid et al., 2016; Chan et al., 2019).

Cataract-related visual damage may result in reduced neuronal input or degeneration of inactive cortical neuronal tissue, accelerating or amplifying neurodegeneration. The visual cortex undergoes structural changes in response to vision loss (von dem Hagen et al., 2005; Boucard et al., 2009). Cataract surgery replaces the previously cloudy lens with a clear IOL, significantly increasing the amount of light reaching the retina. Light stimulation may make the brain more active. Light, as an environmental intervention, can regulate the biological clock. Circadian rhythm disorder makes individuals predispose individuals to cognitive impairment or to the clinical onset of AD. Therefore, increasing the retina's light can also serve to prevent cognitive impairment or AD (Miyata et al., 2018).

Intrinsically photosensitive retinal ganglion cells (ipRGCs) are susceptible to short wavelength (blue) light, and blue light exposure is most effective in improving cognitive performance and enhancing brain responses (von dem Hagen et al., 2005). Light guides people through cognitive tasks visually and exerts non-visual effects. ipRGCs' non-visual responses to light may affect many brain functions, including cognitive functions (Vandewalle et al., 2009). Age-related cataracts have a yellowish nucleus that filters out blue light. Consequently, the stimulating influence of blue light on the ipRGCs, which has been demonstrated to connect with cognitive illnesses, including AD, is another possible cause for why cataract removal is associated with decreased risk of dementia. The elderly may engage more socially if their visual function improves after cataract surgery. Additionally, cataract surgery in the elderly is linked to a decline in depressive symptoms, which may encourage social interaction even more (Walker et al., 2006; Ishii et al., 2008). The link between cataract surgery and dementia has to be further studied.

However, the number of cohort studies included in the meta-analysis was relatively small. There were only four studies, including retrospective studies, which may have recall bias. More prospective research is required to understand the connection better, and the included literature did not provide detailed data on gender and follow-up time points. It, therefore, did not allow for more subgroup analysis.

## Conclusions

Cataract surgery is linked to a lower incidence of all-cause dementia and Alzheimer's disease. A cataract is a reversible visual impairment. Cataract surgery may be a protective factor against the onset of all-cause dementia and can reduce the economic and family burden caused by all-cause dementia worldwide. Given the restricted pool of included studies, our findings necessitate meticulous interpretation.

## Data availability statement

The original contributions presented in the study are included in the article/Supplementary material, further inquiries can be directed to the corresponding author.

## Author contributions

QZ and YJ contributed to design and conception of the study. QZ drafted the manuscript and XW collected the data. WZ and LX conducted an analysis. HR, XL, and ZC contributed to data interpretation and revised the manuscript. XB and RF made critical revisions. All authors have read and approved the manuscript.
