# Supplementary material for: Association of cataract extraction and the risk of dementia—A systematic review and meta-analysis
Source: Front Aging Neurosci. 2023 May 25;15:1168449. doi: 10.3389/fnagi.2023.1168449 (PMC10248513; doi:10.3389/fnagi.2023.1168449)
Supplement: Supplementary file 1 [file Table_1.DOCX]

**Supplementary table 1**

**Details of the Literature Search Strategy**

(1) PubMed

| **Search** | **Query** | **Items found** |
| --- | --- | --- |
| #1 | "Dementia"[MeSH] | 197,300 |
| #2 | "Alzheimer Disease"[MeSH] | 113,495 |
| #3 | ((Dementia*[Title/Abstract]) OR (Amentia*[Title/Abstract])) OR (Alzheimer*[Title/Abstract]) | 263,910 |
| **#4** | #1 OR #2 OR #3  (("Dementia"[Mesh]) OR ("Alzheimer Disease"[Mesh])) OR (((Dementia*[Title/Abstract]) OR (Amentia*[Title/Abstract])) OR (Alzheimer*[Title/Abstract])) | 305,450 |
| #5 | "Cataract Extraction"[Mesh] | 36,576 |
| #6 | (((Cataract Extraction*[Title/Abstract]) OR (Phakectom*[Title/Abstract])) OR (Enzymatic Zonulolys*[Title/Abstract])) OR (Cataract Surgery[Title/Abstract]) | 29,014 |
| **#7** | #5 OR #6  "Cataract Extraction"[Mesh] OR (((Cataract Extraction*[Title/Abstract]) OR (Phakectom*[Title/Abstract])) OR (Enzymatic Zonulolys*[Title/Abstract])) OR (Cataract Surgery[Title/Abstract]) | 45,437 |
| #8 | #4 AND #7  ((("Dementia"[Mesh]) OR ("Alzheimer Disease"[Mesh])) OR (((Dementia*[Title/Abstract]) OR (Amentia*[Title/Abstract])) OR (Alzheimer*[Title/Abstract]))) AND (("Cataract Extraction"[Mesh]) OR (((((Cataract Extraction*[Title/Abstract]) ) OR (Phakectom*[Title/Abstract])) OR (Enzymatic Zonulolys*[Title/Abstract])) OR (Cataract Surgery[Title/Abstract]))) | **96** |

(2) Embase

| No. | Content | Result |
| --- | --- | --- |
| #1 | 'dementia'/exp | 423,619 |
| #2 | 'alzheimer disease'/exp | 234,067 |
| #3 | dementia*:ab,ti OR amentia*:ab,ti OR alzheimer*:ab,ti | 409,674 |
| **#4** | #1 OR #2 OR #3 | 502,198 |
| #5 | 'cataract extraction'/exp | 56,691 |
| #6 | 'cataract extraction*':ab,ti OR phakectom*:ab,ti OR 'enzymatic zonulolys*':ab,ti OR 'cataract surgery':ab,ti | 33,639 |
| **#7** | #5 OR #6 | 61,226 |
| #8 | #4 AND #7 | **218** |

(3) Cochran Library

| No. | Content | Result |
| --- | --- | --- |
| #1 | MeSH descriptor: [Dementia] explode all trees | 6831 |
| #2 | MeSH descriptor: [Alzheimer Disease] explode all trees | 3838 |
| #3 | (Dementia*):ti,ab,kw OR (Amentia*):ti,ab,kw OR (Alzheimer*):ti,ab,kw | 22807 |
| **#4** | #1 OR #2 OR #3 | 23097 |
| #5 | MeSH descriptor: [Cataract Extraction] explode all trees | 2876 |
| #6 | (Cataract Extraction*):ti,ab,kw OR (Phakectom*):ti,ab,kw OR (Enzymatic Zonulolys*):ti,ab,kw OR(Cataract Surgery):ti,ab,kw | 7149 |
| **#7** | #5 OR #6 | 7486 |
| #8 | #4 AND #7 | 14 |
